# Tagging Frogs with Passive Integrated Transponders Causes Disruption of the Cutaneous Bacterial Community and Proliferation of Opportunistic Fungi

**DOI:** 10.1128/AEM.01175-14

**Published:** 2014-08

**Authors:** Rachael E. Antwis, Gerardo Garcia, Andrea L. Fidgett, Richard F. Preziosi

**Affiliations:** a Faculty of Life Sciences, University of Manchester, Manchester, United Kingdom; b Chester Zoo, Upton-by-Chester, United Kingdom

## Abstract

Symbiotic bacterial communities play a key role in protecting amphibians from infectious diseases including chytridiomycosis, caused by the pathogenic fungus Batrachochytrium dendrobatidis. Events that lead to the disruption of the bacterial community may have implications for the susceptibility of amphibians to such diseases. Amphibians are often marked both in the wild and in captivity for a variety of reasons, and although existing literature indicates that marking techniques have few negative effects, the response of cutaneous microbial communities has not yet been investigated. Here we determine the effects of passive integrated transponder (PIT) tagging on culturable cutaneous microbial communities of captive Morelet's tree frogs (Agalychnis moreletii) and assess the isolated bacterial strains for anti-B. dendrobatidis activity *in vitro*. We find that PIT tagging causes a major disruption to the bacterial community associated with the skin of frogs (∼12-fold increase in abundance), as well as a concurrent proliferation in resident fungi (up to ∼200-fold increase). Handling also caused a disruption the bacterial community, although to a lesser extent than PIT tagging. However, the effects of both tagging and handling were temporary, and after 2 weeks, the bacterial communities were similar to their original compositions. We also identify two bacterial strains that inhibit B. dendrobatidis, one of which increased in abundance on PIT-tagged frogs at 1 day postmarking, while the other was unaffected. These results show that PIT tagging has previously unobserved consequences for cutaneous microbial communities of frogs and may be particularly relevant for studies that intend to use PIT tagging to identify individuals involved in trials to develop probiotic treatments.

## INTRODUCTION

Symbiotic bacterial communities have been shown to play a key role in protecting amphibians from infectious diseases such as chytridiomycosis, caused by the virulent and pathogenic Batrachochytrium dendrobatidis fungus (reviewed in reference 1). Bacterial communities associated with the skin of amphibians may protect the host from pathogens (i) by increasing competition for space and resources, (ii) by altering the microenvironment of the amphibian skin to prevent colonization of pathogens, and (iii) through the production of antimicrobial metabolites that kill or inhibit the growth of pathogens (2–4). The potential for such bacteria to act as probiotic treatments for amphibians against chytridiomycosis is currently being investigated (reviewed in reference 1), and ongoing research has identified symbiotic bacteria that inhibit the growth of B. dendrobatidis *in vitro* from a number of amphibian species (5–11).

Given the role of bacterial communities in protecting amphibians from pathogenic infection, events that lead to the disruption of the bacterial community may have implications for the susceptibility of amphibians to disease. Both in the wild and in captivity, amphibians are often marked for a variety of reasons, for example, to identify individuals, to avoid resampling, or to conduct mark-release-recapture surveys. Historically, toe clipping was often used for identification purposes, although recent advances in other, less-invasive techniques such as passive integrated transponder (PIT) tagging or visible implant elastomer (VIE) dyes are now more commonly used. The existing literature indicates that these marking techniques have very few negative effects on amphibians (e.g., 12–19), but they have not yet been investigated for their effects on amphibian cutaneous microbial communities. Here, we determine the effects of PIT tagging on cutaneous microbial communities of captive Morelet's tree frogs (Agalychnis moreletii) and assess the isolated bacterial strains for anti-B. dendrobatidis activity *in vitro*.

## MATERIALS AND METHODS

### Ethics statement.

The Chester Zoo Ethical Committee and the University of Manchester Ethics Committee approved this study prior to its start. This study did not require a license from the United Kingdom's Home Office as PIT tagging is an approved method for the identification of frogs, and data were collected during routine marking of frogs for identification purposes.

### Study animals, husbandry, and experimental design.

A total of 20 adult Agalychnis moreletii frogs were used in this study: 10 (5 males and 5 females) in the nonmarked (control) group and 10 (5 males and 5 females) in the marked group. All frogs were from an F1 generation of a conservation breeding program at Chester Zoo, United Kingdom, and were maintained in 24- by 18- by 18-in. tanks with a drilled base for drainage. Frogs were maintained in groups of four, but only two frogs from each tank were used in this study, with one frog PIT tagged and the other not marked (i.e., not all frogs in each tank were involved in the study). This controlled for variation between individuals according to tank. Tanks contained a water dish and cuttings of devil's ivy (Scindapsus sp.), and frogs were fed crickets gut-loaded on fresh fruit and vegetables and dusted with Nutrobal (VetArk, United Kingdom) two to three times weekly.

### Marking techniques.

Agalychnis moreletii frogs were marked using PIT tags (Nonatec, United Kingdom). This method was chosen as frogs were due to be rehomed at other institutions, and PIT tagging gives individuals unique codes for identification. Gloves were worn throughout marking and were changed between frogs to minimize cross-contamination. During marking the frog was restrained on a flat and stable surface, and the loaded PIT tagging needle was inserted smoothly and quickly under the skin into the tibiofibular leg muscle (G. Garcia and V. Ogilvy, personal communication). The PIT tagging process was standardized and took approximately 1 min per individual. To ensure that any changes in microbial communities could be attributed to PIT tagging, nonmarked (control) frogs were handled using the same methods and for the same amount of time as marked individuals but without the insertion of a PIT tag. All frogs were monitored for 2 weeks after marking for any signs of adverse reaction, of which none were observed.

### Bacterial and fungal culturing.

Microbial communities were collected from the skin of all frogs at 1 day before marking, 1 day after marking, and then 2 weeks postmarking to determine if communities had returned to their original compositions. Microbial communities were collected using similar methods as previously described in Antwis et al. (20). Briefly, frogs were rinsed using sterile water to remove transient bacteria (21). Frogs were then swabbed on their dorsal and ventral surfaces separately using sterile swabs (Eurotubo, Rubi, Spain). The same body area was swabbed for each frog using a standardized method using 20 strokes over each surface. Gloves were worn throughout the swabbing process and were changed between frogs to minimize cross-contamination. Swabs were placed into 1 M NaCl_2_ solution, and serial dilutions were constructed up to a concentration of 10^−2^. Concentrations of 10^−1^ and 10^−2^ were plated out on low-nutrient agar (R2A; Lab M, United Kingdom). Bacterial and fungal colony growth of different morphotypes (based on color, hue, and texture of the colonies) was counted until negligible new growth was observed (10 days after initial plating). Frogs were checked daily throughout the study and for 2 weeks after for any signs of adverse reaction to the swabbing protocol, of which none were observed.

### Molecular methods and bacterial sequencing.

Pure cultures of each bacterial morphotype were streaked out, and colony PCR was conducted to sequence the conserved region of the 16S rRNA gene to identify bacteria. Colony PCRs were performed using Platinum PCR SuperMix (Invitrogen, Life Technologies) according to the manufacturer's instructions using universal primers 27F (5′-GTGCTGCAGAGAGTTTGATCCTGGCTCAG-3′) and 1492R (5′-CACGGATCCTACGGGTACCTTGTTACGACT-3′) (22). rRNA fragments were amplified under the following conditions: 95°C for 2 min, followed by 35 cycles of 94°C for 30 s, 55°C for 30 s, and 72°C for 90 s, with a final extension step of 5 min at 72°C. PCR products were checked for the correct length using gel electrophoresis and then purified with a GenElute PCR Cleanup kit (Sigma-Aldrich). PCR products were sequenced at the DNA Sequencing Facility, University of Manchester, United Kingdom. A consensus sequence was obtained by combining the forward and reverse sequences in DNA Dynamo sequence analysis software (BlueTractorSoftware, Ltd., United Kingdom), which was then blasted against the NCBI database (http://blast.ncbi.nlm.nih.gov/Blast.cgi) to identify each morphotype to genus level. Morphotypes with 99% sequence similarity or greater were considered the same species (23).

### *In vitro* B. dendrobatidis challenges.

B. dendrobatidis global panzootic lineage (GPL) isolate AUL 1.2 (passage five; isolated in Spain in 2010 from Alytes obstetricans) was used to conduct *in vitro* chytrid challenges for this study. The chytrid culture was grown in 1% TGhL (tryptone, gelatin hydrolysate, lactose) liquid medium at 18°C until maximum zoospore production was observed (at around 3 days). Three milliliters of the chytrid culture was spread across the surface of 1% tryptone–1% agar plates and left to dry under a sterile hood, ensuring that plates did not dry out completely but were not too wet as to cause the bacterial streaks to run (5–7, 21). Two bacterial pure cultures were then streaked onto opposing sides of each plate. Plates were inverted and incubated at 18°C for 10 days. Bacterial streaks were then scored for the presence or absence of a zone of inhibition, indicated by markedly reduced or absent growth of chytrid (5–7, 21). If both bacterial streaks on one plate exhibited inhibition, the *in vitro* challenge was repeated for both bacterial strains separately using a noninhibitory bacterial strain as a control.

### Data conversion and statistical analyses.

Bacterial and fungal counts were multiplied by their respective dilution factors and averaged for each morphotype. The total bacterial abundance (total number of cultured bacteria for each individual) and morphotype richness (the number of different morphotypes associated with each individual) were then calculated for each sample, along with the total fungal abundance.

Bacterial abundance data were Poisson distributed, and so the effect of sampling point (1 day premarking, 1 day postmarking, and 2 weeks postmarking), treatment group (marked or control), surface (dorsum or ventrum), gender (male or female), and all possible interactions were analyzed using JMP 10 software with a general linear model (GLM) with a Poisson distribution. All terms were highly significant (see Results), and so the percent change in bacterial abundance from premarking to 1-day postmarking was analyzed in JMP 10 using a GLM including all variables and possible interactions.

The effect of sampling point, treatment group, surface, gender, and all possible interactions on bacterial morphotype richness was analyzed in JMP 10 using a GLM with stepwise selection (using a comparison of the Akaike information criterion [AIC] value), which produced a final model containing surface, gender, and their interaction.

Data were combined for the dorsal and ventral surfaces of each frog, and differences in the overall bacterial community compositions according to treatment and gender were analyzed for each sampling point separately using the Adonis function of the Vegan package in RStudio. The variation in bacterial community structure according to sampling point and treatment group was visualized using nonmetric multidimensional scaling (NMDS) in RStudio. In addition, the relative abundance of each bacterial morphotype was calculated for each treatment group within a sampling point and plotted in GraphPad Prism, version 6.

Combined data for the dorsum and ventrum of each individual were also used to determine changes in the abundances of the two anti-B. dendrobatidis bacterial morphotypes identified in this study (see Results). These data were Poisson distributed, and so the percent change in bacterial abundance from premarking to 1 day postmarking was analyzed in JMP 10 using a generalized linear model with a Poisson distribution including treatment and gender.

Fungal abundance data were Poisson distributed, and so the effect of sampling point, treatment group, surface, gender, and all possible interactions were analyzed in JMP 10 using a generalized linear model with a Poisson distribution.

### Nucleotide sequence accession numbers.

Sequences of the 16S rRNA genes of the following bacteria were deposited in GenBank (accession number): Brevundimonas sp. (KC853150), Chryseobacterium sp. (KC853148), Citrobacter sp. (KC853149), Gordonia sp. (KF444801), Paracoccus sp. strain A (KF444802), Paracoccus sp. strain B (KC853138), Microbacterium sp. strain A (KC853147), Microbacterium sp. strain B (KF444800), and Micrococcus sp. (KF444803).

## RESULTS

### Bacterial communities.

A total of nine bacterial morphotypes were isolated from A. moreletii frogs in this study: Brevundimonas sp. (GenBank accession number KC853150), Chryseobacterium sp. (KC853148), Citrobacter sp. (KC853149), Gordonia sp. (KF444801), Paracoccus sp. strain A (KF444802), Paracoccus sp. strain B (KC853138), Microbacterium sp. strain A (KC853147), Microbacterium sp. strain B (KF444800), and Micrococcus sp. (KF444803).

**FIG 1:**
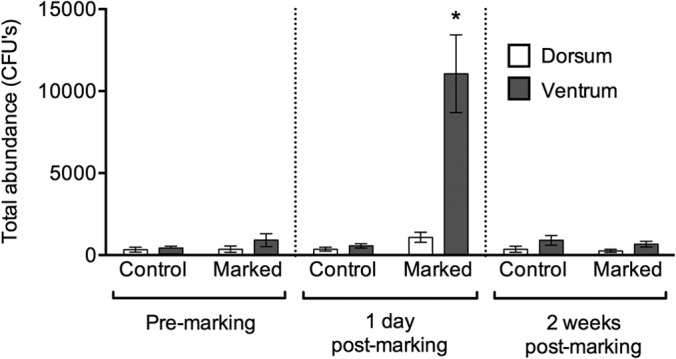
Total abundance of bacteria isolated from the dorsal and ventral surfaces of Agalychnis moreletii frogs either marked using PIT tags (marked) or handled without marking (control). Error bars show ±1 standard error of the mean. *, significantly different result.

The generalized linear model for bacterial abundance was statistically significant (χ^2^ = 355,845.7, df = 23, *P* < 0.0001), with highly significant effects of all variables (sampling point, treatment group, surface, and gender) and interactions (*P* < 0.0001). The GLM for the percent change in bacterial abundance from 1 day premarking to 1 day postmarking was statistically significant (*F*_7,28_ = 3.920, *P* = 0.0042). A Student's *post hoc* analysis showed a significant difference between control and marked frogs (*P* = 0.024) and a significant interaction between treatment and surface (*P* = 0.041), with a significant increase in the bacterial abundance associated with the ventral surfaces of frogs that were marked (∼12-fold) at 1 day postmarking (Fig. 1). There was also a small increase in the abundance of bacteria associated with the dorsal surfaces of marked frogs at this time point in comparison to frogs in the control group (∼3-fold) (Fig. 1).

**FIG 2:**
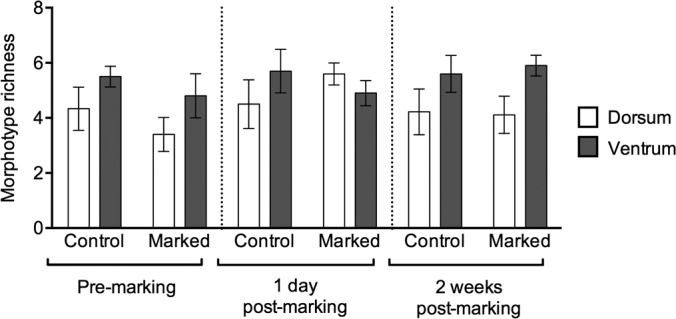
Morphotype richness of bacteria isolated from the dorsal and ventral surfaces of Agalychnis moreletii frogs either marked using PIT tags (marked) or handled without marking (control). Error bars show ±1 standard error of the mean.

Once model reduction had been conducted, the GLM for morphotype richness containing only gender and surface was statistically significant (*F*_3,110_ = 6.486, *P* = 0.0004). Student's *post hoc* analyses showed that the ventral surfaces of frogs supported a significantly greater richness than the dorsal surfaces (*P* = 0.0038) (Fig. 2) and that females supported significantly greater morphotype richness than males (*P* = 0.0097). Although there was no significant effect of sampling point or treatment on morphotype richness, Fig. 2 shows that for each treatment group within a sampling point, the ventral surfaces of frogs consistently supported a greater morphotype richness than the dorsal surfaces, except for marked frogs at 1 day postmarking, for which the morphotype richness of the ventral surfaces was lower than that of the dorsal surfaces.

**FIG 3:**
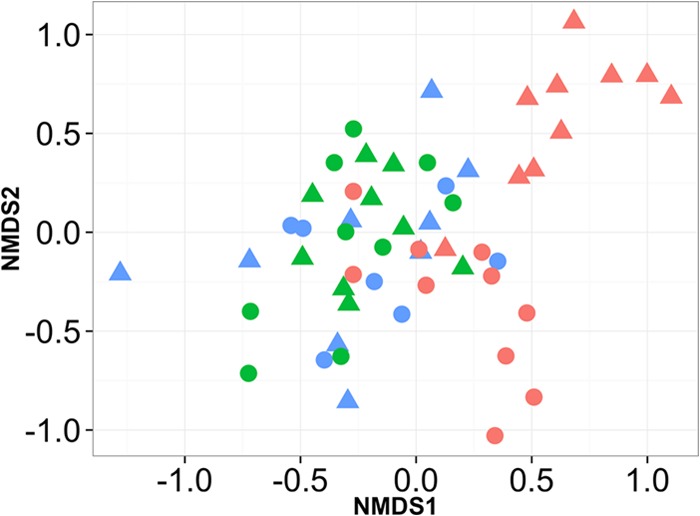
Ordination plot of NMDS (nonmetric multidimensional scaling) analysis of bacterial communities isolated from Agalychnis moreletii frogs either marked using PIT tags (triangles) or handled without marking (circles). Bacterial communities were cultured at 1 day premarking (blue), 1 day postmarking (red), and 2 weeks postmarking (green).

**FIG 4:**
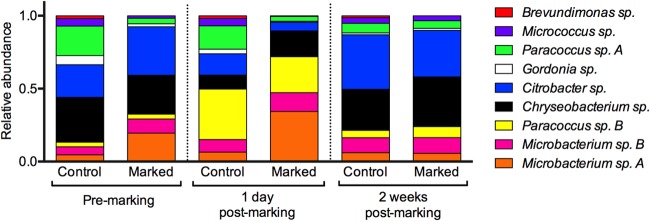
Average relative abundances of bacterial morphotypes isolated from Agalychnis moreletii frogs either marked using PIT tags (marked) or handled without marking (control).

The results of the Adonis analysis showed no significant differences in the overall community compositions at 1 day premarking according to treatment (*F*_1,19_ = 1.516, *P* = 0.148), gender (*F*_1,19_ = 1.299, *P* = 0.195), or their interaction (*F*_2,18_ = 1.171, *P* = 0.302). At 1 day postmarking there was a highly significant difference in the overall composition of the bacterial community associated with frogs according to treatment (*F*_1,19_ = 11.142, *P* = 0.001) and a marginally significant interaction between treatment and gender (*F*_2,18_ = 2.073, *P* = 0.049) but no effect of gender alone (*F*_1,19_ = 1.423, *P* = 0.207). At 2 weeks postmarking, there were no significant differences in the overall bacterial community compositions according to treatment (*F*_1,19_ = 0.633, *P* = 0.678), gender (*F*_1,19_ = 0.903, *P* = 0.516), or their interaction (*F*_2,18_ = 0.926, *P* = 0.506). The ordination plot from the NMDS analysis (Fig. 3) shows that the premarking (blue shapes) and 2-week postmarking (green shapes) bacterial communities for both treatment groups primarily cluster together around the center of the plot, whereas the 1-day postmarking bacterial communities for control frogs (red circles) form a group that is partially separate from the other data, and those for marked frogs (red triangles) are almost entirely separate from the premarking and 2-week postmarking bacterial communities. The relative abundances of bacteria in the communities for the two treatment groups are fairly similar premarking (Fig. 4) although marked frogs had a greater abundance of Microbacterium sp. A (KC853147) and Citrobacter sp. (KC853149), whereas the control frogs supported a greater abundance of other morphotypes such as Gordonia sp. (KF444801), Paracoccus sp. A (KF444802), and Micrococcus sp. (KF444803). Both groups of frogs (marked and control) showed changes in the relative abundances of morphotypes at 1 day postmarking, in particular, an increase in Paracoccus sp. B (KC853138). At 2 weeks postmarking, the relative abundance of morphotypes associated with marked frogs was very similar to that of control frogs (Fig. 4).

### Anti-B. dendrobatidis bacteria.

The *in vitro* challenge assays revealed two bacterial strains that inhibited B. dendrobatidis: Citrobacter sp. KC853149 and Brevundimonas sp. KC853150.

The generalized linear model for change in abundance of Citrobacter sp. KC853149 from premarking to 1 day postmarking was significant (χ^2^ = 207,086.7, df = 3, *P* < 0.0001), with treatment, gender, and their interaction terms all highly significant (*P* < 0.0001). Overall Citrobacter sp. KC853149 was significantly more abundant at 1 day postmarking on frogs that were PIT tagged and for males more so than for females.

The generalized linear model for change in abundance of Brevundimonas sp. KC853150 was not statistically significant (χ^2^ = 0.001, df = 4, *P* = 0.999), indicating that the abundance of this anti-B. dendrobatidis morphotype did not change for either treatment group at 1 day postmarking.

### Fungal communities.

**FIG 5:**
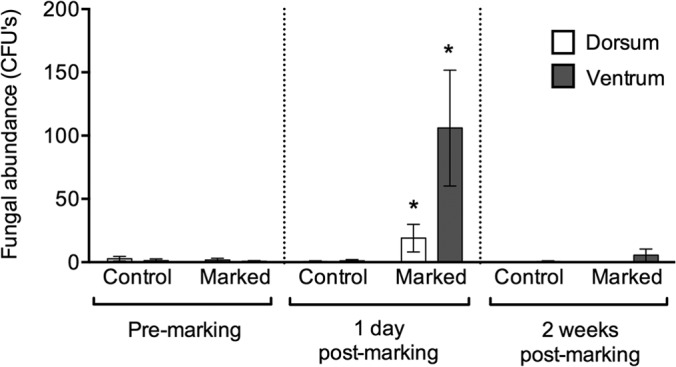
Total abundance of fungi isolated from Agalychnis moreletii frogs either marked using PIT tags (marked) or handled without marking (control). Error bars show ±1 standard error of the mean. *, significantly different result.

The generalized linear model for fungal abundance was highly significant (χ^2^ = 4,360.5, df = 11, *P* < 0.0001). Effect tests show a significant effect of treatment (*P* < 0.0001) and surface (*P* < 0.0001) and a significant interaction between sampling point and treatment (*P* < 0.0001) and between sampling point and surface (*P* < 0.0001). The abundance of fungal colonies isolated from the ventral surfaces of marked frogs at 1 day postmarking was ∼200-fold greater than that at premarking and ∼12-fold greater than that for the dorsal surfaces (Fig. 5).

## DISCUSSION

In this study, we show that PIT tagging Agalychnis moreletii frogs causes an ∼12-fold increase in the abundance of culturable bacteria associated with their ventral surfaces, as well as up to an ∼200-fold increase in the growth of resident fungi (Fig. 1 and 5). In addition, changes in the bacterial community structure and relative abundances of bacterial morphotypes over the course of the study indicate that both control and marked frogs experienced a disruption to their bacterial communities. These results have implications for the use of PIT tagging for the monitoring of wild populations, particularly those involved in field studies for the development of probiotic treatments. Moreover, the disturbance caused to bacterial communities during handling of frogs may complicate studies that investigate changes in bacterial communities over time.

The bacterial communities associated with frogs were not significantly different between marked and control frogs at the start or end of the study, but they were significantly different at 1 day postmarking. The clustering of the bacterial communities in the NMDS ordination plot for the two treatment groups was tightest at 2 weeks postmarking but still overlapping with data points for the bacterial communities associated with all frogs premarking, indicating that the bacterial communities returned to their previous compositions after the “disturbance events” (handling and PIT tagging) (Fig. 3). In addition, by the end of the study the relative abundances of bacterial morphotypes of both groups had reset to more or less similar proportions to one another and to their previous compositions at the start of the study (Fig. 4). This indicates that although the skin microbiota of amphibians may be susceptible to disturbance, amphibians potentially maintain a favored microbiota to which they return. This hypothesis is supported by studies that show that the compositions of bacterial communities are strongly dependent on host species (24, 25), the identification of a core bacterial community that is maintained on removal from the wild to captivity (10), and the resilience of bacterial communities to colonization from other microbiota through the regulation of AMP production (26). More work is required to determine the stability and resilience of the amphibian microbiota to other disturbances, along with mechanisms for regulating the microbiota, and the implications of these for the susceptibility of amphibians to infectious diseases.

Nine bacterial morphotypes were isolated in this study, which is a relatively low number compared to some studies (see, for example, references 6, 7, and 21) but similar to the results of others (see, for example, references 20, 27, 28, and 29). Captive amphibians (as used in this study) have been shown to possess less diverse bacterial communities than their wild counterparts (10, 20), which may account for the relatively low number of morphotypes isolated here. Culturing methods are known to greatly underestimate microbial richness and abundance (reviewed in reference 30), and molecular techniques (e.g., next-generation sequencing) are required to more fully characterize the community. However, the data presented in this study show that PIT tagging had a very strong effect on the culturable microbial community, which is likely to be applicable to the rest of the nonculturable community.

We identified two anti-B. dendrobatidis bacterial morphotypes resident on the skin of A. moreletii. One of these strains (Citrobacter sp. KC853149) significantly increased in abundance at 1 day postmarking for frogs that were PIT tagged although this might be expected, given the significant increase in bacterial abundance overall for PIT-tagged frogs (Fig. 1). The other anti-B. dendrobatidis strain (Brevundimonas sp. KC853150) was not affected by treatment or sampling point during the study although this was a rare bacterial morphotype and so may have been relatively unaffected by the handling and PIT tagging (Fig. 4). There are likely to be other anti-B. dendrobatidis bacteria in the nonculturable portion of the microbiota on the skin of this amphibian species, and the effect of PIT tagging on these is unknown. Further studies that determine whether PIT tagging affects the susceptibility of amphibians to infectious diseases, including chytridiomycosis, are required.

The mechanism that causes these changes in the bacterial communities of marked frogs (and to a lesser degree, control frogs) is not clear. The act of tagging (and to a lesser degree, handling) may have led to changes in hormone production or other biochemical signals (e.g., glucocorticoids, catecholamines, etc.) that altered the host biology (e.g., peptide secretion) or interacted directly with the bacterial community (31–39). More work is needed to determine the effects of marking techniques on glucocorticoid production and peptide secretion and their subsequent effects on bacterial communities in amphibians. It is unlikely that the PIT tag itself introduced microbes that subsequently proliferated on the skin of the frog since the bacterial morphotypes isolated at 1 day postmarking were the same as throughout the rest of the study, since the PIT tags and syringes were sterile and individually packaged, and since there were also changes observed in the bacterial communities of control frogs.

A number of the results presented here indicate differences between genders (i.e., morphotype richness, bacterial abundance, changes in abundance of anti-B. dendrobatidis bacteria). This may be due to the much greater snout-vent length of females than of males of A. moreletii, meaning that there was a greater surface area for microbes to attach. Gender differences were not seen in the closely related Agalychnis callidryas frogs (20), in which females are still larger than males but to a lesser degree than in A. moreletii (R. Antwis, personal observation). Future studies may need to take into account potential differences in microbial communities between genders (due to morphometrics or physiological factors), particularly in the development of probiotic treatments.

The ventral surfaces of frogs that were PIT tagged experienced a much greater disturbance in the microbiome than the dorsal surfaces in terms of an increase in both bacterial and fungal abundances. This may be due to the greater initial abundance of bacteria (and to some extent, fungi) on the ventrum than the dorsum, possibly arising from the greater fluxes of water and solutes experienced across the ventral surfaces of frogs, leading to richer and more abundant bacterial communities (40), as well as from the greater contact between the ventral surfaces and the substrates, leading to greater exposure to microbes in the environment. In addition, mucous and serous glands are asymmetrically distributed across the skin of Phyllomedusinae frogs (40–42), with higher concentrations of serous glands on the dorsal surfaces and greater abundances of mucous glands on the ventral surfaces (43–45). The biochemicals (e.g., peptides and glycosaminoglycans) produced by both gland types are likely to influence the bacterial community associated with either surface of the skin of frogs (40–45). Greater mucus production on the ventrum may encourage bacterial growth, whereas greater peptide production on the dorsum may inhibit it.

In conclusion, PIT tagging causes a major disruption to the bacterial community associated with the skin of frogs, as well as a concurrent proliferation in resident fungi. This may be particularly relevant for studies that intend to use PIT tagging to follow individual animals involved in field studies for probiotic treatments, as changes in bacterial communities may be confounded by the method used to mark amphibians. Investigation into the mechanism that drives this phenomenon is warranted, in addition to studies that determine whether PIT tagging increases the susceptibility of amphibians to infectious diseases. Furthermore, studies are required to determine the effects of other marking techniques on the bacterial communities of amphibians.
